# Evaluating the protective effect of resveratrol, Q10, and alpha-lipoic acid on radiation-induced mice spermatogenesis injury: A histopathological study

**DOI:** 10.18502/ijrm.v17i12.5791

**Published:** 2019-12-30

**Authors:** Masoud Najafi, Mohsen Cheki, Peyman Amini, Abdolreza Javadi, Dheyauldeen Shabeeb, Ahmed Eleojo Musa

**Affiliations:** ^1^ Department of Radiology and Nuclear Medicine, School of Paramedical Sciences, Kermanshah University of Medical Sciences, Kermanshah, Iran.; ^2^ Toxicology Research Center, Ahvaz Jundishapur University of Medical Sciences, Ahvaz, Iran.; ^3^ Department of Radiology, Faculty of Paramedical, Tehran University of Medical Sciences, Tehran, Iran.; ^4^ Department of Pathology, Imam Hossein Hospital, Shahid Beheshti University of Medical Sciences, Tehran, Iran.; ^5^ Department of Physiology, College of Medicine, University of Misan, Misan, Iraq.; ^6^ Research Center for Molecular and Cellular Imaging, Tehran University of Medical Sciences, Tehran, Iran.

**Keywords:** Radiation, Spermatogenesis, Resveratrol, Alpha-lipoic acid, Coenzyme Q10.

## Abstract

**Background:**

Testis is one of the most sensitive organs against the toxic effect of ionizing radiation. Exposure to even a low dose of radiation during radiotherapy, diagnostic radiology, or a radiological event could pose a threat to spermatogenesis. This may lead to temporary or permanent infertility or even transfer of genomic instability to the next generations.

**Objective:**

In this study, we evaluated the protective effect of treatment with three natural antioxidants; resveratrol, alpha lipoic acid, and coenzyme Q10 on radiation-induced spermatogenesis injury.

**Materials and Methods:**

30 NMRI mice (6-8 wk, 30 ± 5 gr) were randomly divided into six groups (n = 5/each) as 1) control; 2) radiation; 3) radiation + resveratrol; 4) radiation + alpha lipoic acid; 5) radiation + resveratrol + alpha lipoic acid; and 6) radiation+ Q10. Mice were treated with 100 mg/kg resveratrol or 200 mg/kg alpha lipoic acid or a combination of these drugs. Also, Q10 was administered at 200 mg/kg. All treatments were performed daily from two days before to 30 min before irradiation. Afterward, mice were exposed to 2 Gy ^60^Co gamma rays; 37 days after irradiation, the testicular samples were collected and evaluated for histopathological parameters.

**Results:**

Results showed that these agents are able to alleviate some toxicological parameters such as basal lamina and epididymis decreased sperm density. Also, all agents were able to increase Johnsen score. However, they could not protect against radiation-induced edema, atrophy of seminiferous tubules, and hyperplasia in Leydig cells.

**Conclusion:**

This study indicates that resveratrol, alpha-lipoic acid, and Q10 have the potential to reduce some of the side effects of radiation on mice spermatogenesis. However, they cannot protect Leydig cells as a source of testosterone and seminiferous tubules as the location of sperm maturation.

## 1. Introduction

The testis is one of the most sensitive organs against the toxic effects of clastogenic agents such as metals, chemotherapy agents, as well as non-ionizing and ionizing radiation (IR) (1). Seminiferous tubules, which are located within the testis comprises Sertoli cells that supply hormonal development and differentiation of germ cells to form new and mature sperms. Seminiferous tubules are very sensitive to toxic agents and oxidative stress that may suppress the formation of new spermatozoa and affect reproduction (2). Germ cells within the seminiferous tubules can be potently affected even by low-dose radiation. A dose of IR equal to 0.3 Gy or more can lead to temporary or permanent infertility (3). Experimental studies have indicated that exposure of rats or mice to 1-2 Gy causes a significant reduction in sperm contents and morphological damages (4). Exposure to a sub lethal dose of IR during a radiation disaster and radiological events, in addition to external radiotherapy and radio-iodine therapy, may affect spermatogenesis potently (3). Exposure of testis to IR leads to apoptosis in highly radiosensitive cells such as germ cells, spermatogonia, spermatids, spermatocytes and sperms. This is associated with reduced numbers of these cells some weeks after exposure (5). Also, a low dose of IR may cause mutation in sperm DNA which may be transferred to the next generations. It has been shown that direct exposure of testis to IR, as well as sperm damage by non-targeted effect of IR, causes changes in methylation and some other epigenetic properties that affect carcinogenesis risk in offspring (6).

It is well-known that in addition to the direct damage of IR, through the production of free radicals, it causes mutation and cell death in cells. In addition to produced ROS by IR, it seems that activation of reduction/oxidation (redox) metabolisms plays a key role in apoptosis and cell death. IR can attenuate antioxidant enzymes as well as promote the production of free radicals by pro-oxidant enzymes that amplify radiation toxicity in irradiated organs. Thus, neutralization of free radicals can reduce toxicity and also attenuate redox activity as well as antioxidant suppression following exposure to IR. So far, several experimental studies have been conducted to protect spermatogenesis against IR (7, 8). Experiments have confirmed that some antioxidants and radioprotectors are able to alleviate radiation toxicity in testis (9, 10). Protection of spermatogenesis, as well as other procedures in highly radiosensitive organs, is a substantial rational for the detection of ROS scavengers for accidental or therapeutic exposure to IR (7).

In the current study, we detected possible protective effects of resveratrol, alpha-lipoic acid, and Q10 on radiation-induced testis injury.

## 2. Materials and Methods

### Experimental design

A total of 30 NMRI mice (weighing 30 ± 5 gr) were used throughout the study. All of them were kept in the same room under constant temperature (22 ± 2°C), humidity (55-60%), and illumination (7:00 a.m. to 7:00 p.m.), with free access to food pellets and water. In this experimental study, all mice were randomly divided to six groups (n = 5/each) as follows; group 1 (Control): control without any irradiation or drug; group 2 (RAD): irradiation only with 2 Gy; group 3(RAD+RES): resveratrol+ radiation; group 4(RAD+ALA): alpha lipoic acid+ radiation; group 5(RAD+ALA+RES): radiation+ resveratrol+ alpha lipoic acid; and group 6(RAD+Q10): Q10+radiation. Thirty-seven days after irradiation, all mice were sacrificed. Their right testes were extracted following surgical dissection of their abdomens and immediately fixed in 10% normal buffer formalin.

### Drug treatment and irradiation 

Resveratrol and alpha lipoic acid were purchased from Nanokimia company, Tehran, Iran. Resveratrol was dissolved in 20% ethanol while alpha-lipoic acid was dissolved in distilled water. The concentrations of resveratrol and alpha-lipoic acid were 3 mg/ml and 6 mg/ml, respectively. Moreover, a combination made up of 3 mg/ml resveratrol and 6 mg/ml alpha-lipoic acid was prepared. For treatment with Q10, a capsule containing 100 mg Q10 was dissolved in distilled water at a concentration of 6 mg per ml. Each mouse in the group was treated with 200 mg/kg Q10 orally. InRAD+RES group, mice received 1 ml resveratrol solution equaling 100 mg/kg. RAD+ALA group: mice received 1 ml alpha-lipoic acid solution equaling 200 mg/kg. RAD+ALA+RES group: mice received 1 ml combination form of a solution equaling 100 mg/kg resveratrol and 200 mg/kg alpha-lipoic acid. RAD+Q10 group: micereceived 1ml Q10 solution equaling 100 mg/kg. The dose of Q10 selected was similar to a previous study by Ramadan and colleagues that showed protective effect of this dose against testicular toxicity of magnetic field in mice (11). The dosages of alpha-lipoic acid and resveratrol were chosen similar to some previous studies that showed remarkable normal tissue protection without toxicity (12, 13). All treatments were done orally at one dose per day beginning from two days before irradiation. The last doses of drugs were received 30 mins before irradiation. Prior to irradiation, for anesthesia and fixation of mice, all animals received an intraperitoneal injection of 80 mg/kg ketamine 10% and 5 mg/kg xylazine 2%. After anesthesia, mice were irradiated to the whole body using a ^60^Co gamma ray source. Irradiation was done at a source-to-surface distance (SSD) of 80 cm at a dose rate of 50 cGy/min.

### Histopathological evaluation

Fixed testicular tissues were embedded in paraffin for preparing the slides. Afterward, sections were cut in 4-micron thickness. All sections were stained with hematoxylin and eosin (H&E) for general evaluation of morphological changes. Slides were evaluated with the aid of a light microscope with a magnification of × 100 and × 400. Histological evaluation included different parameters as well as Johnson scoring as explained by Fatehi and colleagues (14). Leydig cell hyperplasia was scored as 0 = normal: no detectable changes in the variability of Leydig cells; 1 = mild changes in the variability of Leydig cells; 2 = enlargement of Leydig cells; and 3 = enlargement and detectable hyperplasia in Leydig cells. Injury to the epididymis was scored as 0 = when lower than 10% tubules affected; 1 = 10-25% affected; 2 = 25-50% affected; and 3 = more than 50% affected. Edema, vacuolation, thickening of basal lamina and epididymis were scored using the same method. Arrest in the spermatogenesis was scored as 0 = normal; 1 = tubular sclerosis; 2 = depletion of germ cells within seminiferous tubules; and 3 = arrest in the spermatocyte stage.

### Ethical consideration

All animal experiments were carried out in accordance with the NIH Guide for Care and Use of Laboratory Animals. All study protocols were approved by the Institutional Animal Ethics Committee of Jundishapur University of Medical Sciences, Ahvaz, Iran (IR.AJUMS.REC.1397.095).

### Statistical analysis

All results were processed using the Statistical Package for the Social Sciences, version 21.0, SPSS Inc., Chicago, Illinois, USA. Results of histopathological changes were analyzed by pair-wise comparison with the Mann-Whitney test. Data were calculated as mean ± standard deviation. The differences between means were considered statistically significant when p < 0.05.

## 3. Results 

**Figure 1:**
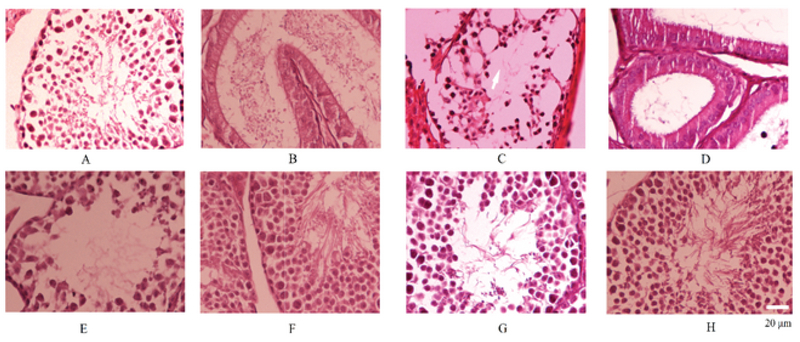
Radioprotective effect of Q10, resveratrol, alpha-lipoic acid, and combination of resveratrol and alpha-lipoic acid on mice spermatogenesis. A and B: normal spermatogenesis; C: reduction of spermatogonia cells; D: spermatogenic arrest in epididymis; E: Radiation + Q10; F: Radiation + Resveratrol; G: Radiation + Alpha-lipoic acid; and H: Radiation + Resveratrol + Alpha-lipoic acid. Irradiation caused significant reduction of spermatogonia numbers, while treatment with Q10, resveratrol, alpha-lipoic acid, or combination of resveratrol and alpha-lipoic acid was able to reduce radiation suppressive effect (H&E staining, Nikon E400 Camera DSFi1, ×40 magnification for A, C, E, F, G, and H, and ×400 magnification for B and D).

As shown in Table I, exposure to 2 Gy gamma rays led to significant spermatogenic arrest (in all irradiated mice in radiation group) (p = 0.003), increase in atrophy of seminiferous tubules (p = 0.004), thickening of basal lamina (p = 0.004), Leydig cells hyperplasia (p = 0.004), edema (p = 0.005), epididymis decreased sperm density (p = 0.003), epididymis vacuolation (p = 0.004) and reduction of Johnsen score (p = 0.005). Treatment with Q10 could attenuate spermatogenic arrest (p = 0.005), thickening of basal lamina (p = 0.028) and epididymis decreased sperm density (p = 0.005), as well as increased Johnsen score (p < 0.005). Treatment with resveratrol reduced spermatogenic arrest (p = 0.003) and attenuated some other parameters such as thickening of basal lamina (p = 0.004), epididymis decreased sperm density (p = 0.018), and vacuolation (p = 0.014). Also, resveratrol could increase Johnsen score (p < 0.005). Interestingly, atrophy of seminiferous tubules was increased by resveratrol administration (p < 0.013). Similar to Q10, treatment with alpha-lipoic acid could reduce spermatogenic arrest (p = 0.003) and protected against radiation-induced thickening of basal lamina (p = 0.004) and epididymis decreased sperm density (p = 0.003). Also, it could increase Johnsen score (p < 0.005). Results of the combination form of alpha-lipoic acid and resveratrol had a similar effect as resveratrol alone except for epididymis vacuolation. This combination was unable to protect against IR-induced epididymis vacuolation. All drugs were able to attenuate the reduction of germ cells caused by IR (Table I, Figure 1).

**Table 1:** The results of drugs treatment with Q10, resveratrol, and alpha-lipoic acid on radiation-induced mice spermatogenesis injury


	**Control**	**RAD**	**RAD+Q10**	**RAD+RES**	**RAD+ALA**	**RAD+ALA+RES**
Spermatogenic arrest	0 ± 00	1 ± 00 ^*a*^	0 ± 00^*b*^	0 ± 00^*b*^	0 ± 00^*b*^	0 ± 00^*b*^
Atrophy of seminiferous tubules	0 ± 00	1.2 ± 0.40^*a*^	2.33 ± 0.94	2.75 ± 0.43^*b*^	1.75 ± 0.43	2.2 ± 0.40^*b*^
Thickening of basal lamina	0 ± 00	2.2 ± 0.40^*a*^	1.33 ± 0.47^*b*^	1.00 ± 00^*b*^	1.00 ± 00^*b*^	1.2 ± 0.40^*b*^
Leydig cells hyperplasia	0 ± 00	2.2 ± 0.40^*a*^	2.00 ± 0.81	3.00 ± 00	2.66 ± 0.50	2.50 ± 0.50
Edema	0 ± 00	1.5 ± 0.5^*a*^	2.00 ± 0.81	2.25 ± 0.83	1.00 ± 00	2.50 ± 0.86
Epididymis (decreased sperm density)	0 ± 00	4 ± 00^*a*^	2.66 ± 0.47^*b*^	3.00 ± 0.70^*b*^	2.00 ± 1.24^*b*^	3.00 ± 00^*b*^
Epididymis vacuolation	0 ± 00	2.8 ± 0.40^*a*^	2.66 ± 0.47	2.00 ± 00^*b*^	1.25 ± 0.81^*b*^	2.75 ± 0.43
Johnsen score	9 ± 00	4.00 ± 0.63^*a*^	6 ± 00^*b*^	6 ± 00^*b*^	6 ± 00^*b*^	6 ± 00^*b*^
Numbers of spermatogonia per each tubule	172.16 ± 34.1	90 ± 5^*a*^	137.66 ± 21^*b*^	138 ± 8.27^*b*^	138.5 ± 8.13^*b*^	147 ± 2.82^*b*^
Data presented as Mean ± SD, (^*a*^: significant changes compared to the control group; ^*b*^: significant changes compared to radiation group; Mann-Whitney non-parametric test)						
RAD: Radiation group; Q10: Treatment with Coenzyme Q10; RES: Treatment with resveratrol; ALA: Treatment with alpha-lipoic acid

## 4. Discussion

In this study, we aimed to evaluate the radioprotective effect of Q10 against toxic effects of IR on mice spermatogenesis system as well as the radioprotective effect of Q10, resveratrol, and alpha-lipoic acid on the radiation-induced testicular injury. Results showed that exposure of mice to 2 Gy gamma rays led to massive damage to spermatogenesis, seminiferous tubules, basal lamina, Leydig cells, and epididymis. Also, irradiation caused significant edema, decreased sperm density and reduced Johnsen score as an index of healthy spermatogenesis. Treatment with Q10, resveratrol, alpha-lipoic acid, or a combination of resveratrol and alpha-lipoic acid could alleviate some toxic effects of IR, but not all of them. Radiation-induced spermatogenic arrest was mild and suppressed by all three agents. Also, all three antioxidants were able to attenuate thickening of the basal lamina and decreased epididymis sperm density and increased Johnsen score. Epididymis vacuolation was alleviated by resveratrol and alpha-lipoic acid, and also by a combinational form of them. Any of these agents were able to reduce edema, atrophy of seminiferous tubules, and hyperplasia in Leydig cells.

IR has a potentially detrimental effect on spermatogenesis via induction of apoptosis in germ cells and spermatogonia (3). Even a sub-lethal dose of IR, for example, 1-4 Gy, can cause either temporary or permanent infertility (3). Hence, exposure to an accidental nuclear or radiological event may lead to potent inhibition of the reproductive system. Moreover, during radiotherapy of tumors which are adjacent to the testis, exposure to scattered radiation may cause massive damage to spermatogenesis. In addition to infertility, exposure of testis to IR is a threat to carcinogenesis in future generations (15). In-vitro and in-vivo studies have shown body of evidence for delayed genomic instability in the progeny of irradiated cells (16). These body of evidence indicate that IR is able to induce cancer markers in the next generations of irradiated cells. In addition, in an animal model, it has been shown that irradiation of testis or even other organs can induce genomic instability in the germline, which increases the activity of oncogenes in future generations (17). Moller and colleagues showed an increase in aberrant morphology in the sperms of Chernobyl survivors. This study showed that increased aberrant morphology has a direct relationship with the serum levels of antioxidants (18). Abnormal changes in sperm function, chromosomal aberrations, and epigenetic changes have been revealed among radiation workers (19). As a result of the high sensitivity of the testis, several studies, so far, have tried to introduce potent radioprotectors for spermatogenesis.

To date, a large number of studies have been conducted to explore effective agents for amelioration of radiation-induced spermatogenesis injury. Some antioxidant agents such as green tea, ascorbic acid, and alpha-tocopherol have shown abilities to protect the testis against IR (10, 20). Pre-treatment with ascorbic acid and famotidine has shown potent protection against radiation-induced reduction of sperm count and damage to seminiferous tubules (7). In recent years, some studies have shown radioprotective effect of some natural agents in the testis. A study by Marzban *et al*. showed that silymarin as an herb-derived agent is able to reduce apoptosis in sperms, increase Johnson's scoring and number of spermatogonia, and ameliorate Leydig cells injury and damage to seminiferous epithelium (21). Silymarin has also been shown to reduce DNA damage and testicular injury via neutralization of free radicals and reduction of oxidative stress (14). Similar results have been shown for some other natural antioxidants such as melatonin and dimethylaminoparthenolide (DMAPT) (22, 23).

In the present study, we showed that resveratrol, alpha-lipoic acid, and Q10 may protect spermatogenesis against toxic effects of IR. Resveratrol and alpha-lipoic acid are two potent antioxidants and redox modulatory agents that have shown good radioprotective effects. Resveratrol is a herbal substance which can be produced by several plants when they are under attack by pathogens. Evidence show that resveratrol can protect different organs against diseases such as diabetes, heart diseases, and cancer (24). It seems that a substantial effect of resveratrol is mediated through activation of *Sirt1*, a gene which is involved in DNA repair following exposure of cells to clastogenic agents (25). Moreover, studies show that resveratrol can attenuate inflammatory mediators such as NF-κB and cyclooxygenase-2 (COX-2), which are involved in the toxic effects of radiation or other toxic agents via stimulation of free radical production (26). In contrast to resveratrol, alpha-lipoic acid is a direct antioxidant and has the ability to neutralize free radicals through recycling of ascorbic acid and alpha-tocopherol (27). Coenzyme Q10 (also known as ubiquinone) is a natural antioxidant that is found in the inner layer of mitochondria. It has been shown that Q10 through neutralization of mitochondria-derived superoxide and modulation of mitochondrial respiration attenuates oxidative injury. Moreover, via inhibition of pro-apoptotic mediators such as cytochrome C and caspase-9, it can reduce apoptosis (28). Q10 has the ability to increase antioxidant potency of cells through modulation of ascorbic acid and alpha-tocopherol recycle (29).

Resveratrol, alpha-lipoic acid, and Q10 are natural and low-toxic antioxidants that have shown appropriate radioprotection in some organs. Previous studies have shown that resveratrol through stimulation of *Sirt1* gene expression enhances DNA repair ability against clastogenic agents (30). Also, resveratrol is able to stimulate antioxidant enzymes and suppresses pro-oxidant enzymes such as NADPH oxidase, leading to alleviation of radiation injury (31). Alpha-lipoic acid has shown abilities to reduce radiation-induced apoptosis and histopathological injuries in mice small intestine, attenuate mucositis, and salivary glands injury, as well as upregulation of pro-inflammatory cytokines in the thyroids of rats (32). Furthermore, both resveratrol and alpha-lipoic acid are able to mitigate radiation-induced morphological changes in the lung, a radiosensitive and late-responding organ to IR (12). Q10 has also shown abilities to ameliorate radiation nephropathy and cerebral ischemia/reperfusion injury in animal models (33).

## 5. Conclusion 

This study showed that resveratrol, alpha-lipoic acid, and Q10 are able to reduce some side effects of IR on mice spermatogenesis. The most obvious protective effects of these agents were observed for the epididymis, basal lamina and sperms. However, these agents could not protect Leydig cells and seminiferous tubules. Since Leydig cells are sources of testosterone secretion (which is essential for sperm maturation) and seminiferous tubules as the location of sperm maturation, it seems that these antioxidants are not able to prevent or ameliorate radiation suppressive effects on sperm maturation.

##  Conflict of Interest

The authors declare that there are no conflicts of interest.
